# Exploring the Intracrine Functions of VEGF-A

**DOI:** 10.3390/biom11010128

**Published:** 2021-01-19

**Authors:** Sophie Wiszniak, Quenten Schwarz

**Affiliations:** Centre for Cancer Biology, University of South Australia and SA Pathology, Adelaide, SA 5000, Australia; sophie.wiszniak@unisa.edu.au

**Keywords:** VEGF, VEGF-A, intracrine, autocrine, intracellular, signaling, cell survival, apoptosis, cancer, anti-VEGF therapy

## Abstract

Vascular endothelial growth factor A (VEGF-A or VEGF) is a highly conserved secreted signalling protein best known for its roles in vascular development and angiogenesis. Many non-endothelial roles for VEGF are now established, with the discovery that VEGF and its receptors VEGFR1 and VEGFR2 are expressed in many non-vascular cell-types, as well as various cancers. In addition to secreted VEGF binding to its receptors in the extracellular space at the cell membrane (i.e., in a paracrine or autocrine mode), intracellularly localised VEGF is emerging as an important signalling molecule regulating cell growth, survival, and metabolism. This intracellular mode of signalling has been termed “intracrine”, and refers to the direct action of a signalling molecule within the cell without being secreted. In this review, we describe examples of intracrine VEGF signalling in regulating cell growth, differentiation and survival, both in normal cell homeostasis and development, as well as in cancer. We further discuss emerging evidence for the molecular mechanisms underpinning VEGF intracrine function, as well as the implications this intracellular mode of VEGF signalling may have for use and design of anti-VEGF cancer therapeutics.

## 1. Introduction

### 1.1. VEGF Protein Family

Vascular endothelial growth factor A (VEGF-A) is a highly conserved secreted signalling protein and founding member of the VEGF protein family, which additionally includes VEGF-B, VEGF-C, VEGF-D, VEGF-E, and PlGF (Placental Growth Factor) [1]. VEGF-A is best known for its roles in vascular development. While a clear biological role for VEGF-B is yet to be defined, VEGF-C and VEGF-D are strongly implicated in lymphangiogenesis. VEGF-E is encoded by the *Orf* parapoxvirus and plays important roles in virus infection and associated pathology. PlGF plays roles in angiogenesis and can form functional heterodimers with VEGF-A [1].

### 1.2. VEGF-A Isoforms

VEGF-A consists of four major isoforms that are expressed in a tissue-specific and temporal-specific manner. In human, these isoforms are termed VEGF121, VEGF145, VEGF165, and VEGF189, numerically representing the number of amino acids in each of the mature isoforms. Variation in protein length arises from alternative splicing of the eight exons comprising the *Vegfa* gene [2]. While all transcripts contain exons 1–5 and 8, diversity generated through alternative splicing of exons 6 and 7 alter the proteins capacity to bind different receptors and extracellular matrix (ECM) components such as heparan sulphate proteoglycans. Accordingly, while VEGF121 is freely diffusible within the ECM, matrix binding properties of the other isoforms increase according to length. This is thought to establish spatial gradients of VEGF-A focused on the cells from which it is expressed, that are important for its ability to regulate almost all facets of vascular morphogenesis [2,3].

### 1.3. VEGF Receptors

All of the VEGF-A isoforms bind to members of the VEGF tyrosine kinase receptor (VEGFR) family on the surface of vascular endothelial cells [4,5]. While VEGF-A binds with high affinity to both VEGFR1 and VEGFR2, each interaction regulates contrasting outcomes. VEGFR2 is the major mediator of the mitogenic, migratory, permeability, and pro-survival-enhancing effects of VEGF-A. Upon VEGF-A binding to VEGFR2 several tyrosine residues within the cytoplasmic domain are autophosphorylated leading to activation of specific intracellular signalling pathways that mediate functional outcomes. In contrast, the cellular functions mediated by VEGF-A binding to VEGFR1 are a topic of much debate [6]. Upon VEGF-A binding to VEGFR1 there is only weak autophosphorylation within the tyrosine kinase domains, suggesting that it may not be a signalling receptor. Indeed, this has underpinned the suggestion that VEGFR1 may act as a decoy receptor to fine-tune the activities of VEGF-A on the vascular endothelium. The existence of an alternatively spliced soluble VEGFR1 that binds and inhibits VEGF-A induced VEGFR2 activity adds support to the notion that this receptor acts in a decoy manner [7]. In addition to binding to the VEGF TK receptors, VEGF-A isoforms featuring exon 7 also bind to the single pass transmembrane receptors Neuropilin 1 (NRP1) and NRP2. NRPs have very short intracellular domains and are unable to induce cellular signalling in the absence of signalling receptors [8]. In the vasculature, NRP1 is thought to enhance the binding and signalling properties of the VEGF165–VEGFR2 interaction. In other contexts such as neurons, NRPs interact with A-type Plexin receptors to mediate functions of alternative extracellular ligands from the class 3 Semaphorin (SEMA3) family. The necessity of the short, yet highly conserved cytoplasmic domain of NRP1 in promoting arterial and venous patterning in the eye [9] also suggests that this receptor family may have additional functions to their roles as co-receptors for VEGFR and A-type Plexins.

### 1.4. VEGF-A Function

Outside of the vasculature, there are many examples of VEGF-A playing important roles in cellular physiology. Soon after its identification it was realised that VEGF-A could influence both haematopoiesis and the behaviour of white blood cells [10], a feature that seems to be conserved across all species including drosophila [11]. Within the nervous system, VEGF-A directly regulates neuronal migration and axon guidance independent of its roles in blood vessels [12,13]. In addition, VEGF-A has been suggested to play many pleiotropic roles in pathological settings, such as cancer and neurodegeneration.

**Figure 1:**
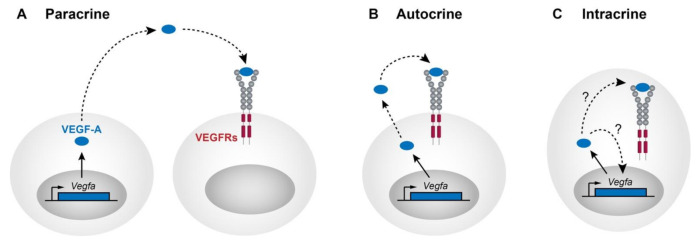
Different modes of Vascular endothelial growth factor A (VEGF-A) signalling. (**A**) VEGF-A characteristically signals through paracrine mechanisms. VEGF-A is synthesised with a secretion signal within signal sending cells and secreted into the extracellular matrix (ECM) as covalently linked homodimers. VEGF-A mediates many cellular responses on distant cells through interactions with several types of receptors including VEGFR1, VEGFR2, and also NRPs (not pictured). (**B**) After being secreted from cells, VEGF-A has also been shown to control many cellular responses through binding to receptors in an autocrine manner. (**C**) Emerging evidence suggests that VEGF-A can signal, without being secreted, in an intracrine manner. In different cell types and in different contexts this mode of signalling may proceed through interactions with receptors located within the cell, through relocalisation to the nucleus, or through mechanisms yet to be fully characterised.

The gap in knowledge leading to discovery of VEGF-A was driven by the desire to identify a diffusible factor that could regulate vascular permeability [14,15] and act as a mitogen for endothelial cells in culture [16]. This line of investigation inherently led research toward its paracrine functions on the blood vasculature (Figure 1A), further strengthened by the finding that all known isoforms of VEGF-A contain an N-terminal signal sequence which promotes secretion of the mature protein in the form of covalently linked homodimers. In addition to its paracrine roles, there are now clear roles for VEGF-A acting in an autocrine manner (Figure 1B). Amongst other examples, VEGF-A is expressed and secreted by endothelial cells to promote homeostasis of blood vessels in the adult [17]. Together, the analysis of autocrine and paracrine functions inform much of our current understanding of the mechanisms through which VEGF-A functions. However, in contrast to its roles outside of the cell where interactions with cell surface receptors drive signalling pathways, there are emerging functions for VEGF-A acting within the cell that it is expressed, in an intracrine manner (Figure 1C). Here we review the current literature supporting a role for VEGF-A as an intracrine (summarised in Table 1), discuss shortfalls in our mechanistic understanding of this phenomena, and propose some simple lines of investigation to expand our knowledge of VEGF-A intracrine signalling.

## 2. Intracrine VEGF in Tissue Development and Homeostasis

### 2.1. Endothelial Cell Migration

One of the first suggestions that VEGF could function intracellularly, in addition to binding VEGF receptors on the extracellular cell membrane, came from the finding that fluorescently-labelled VEGF165 protein became localised to the nucleus of cultured endothelial cells following scratch-wounding [18]. This nuclear localisation was dependent on the ability of VEGF to bind VEGFR2, but nuclear accumulation occurred rapidly and independent of the classical clathrin-coated endocytic-vesicle pathway, suggesting an alternative route. Nuclear accumulation of VEGF in cells situated at the edge of scratch wound correlated with increases in tissue factor (TF) and Factor VIII, which are typical of the wound healing response, and these increases were blocked when nuclear VEGF translocation was inhibited with neutralising antibodies. The authors postulated that stretches of basic amino acids in VEGF may act as a nuclear localisation sequence (NLS), but proof of VEGF function as a nuclear messenger awaits demonstration that NLS-mutant forms of VEGF fail to induce TF and FVIII upon wounding.

### 2.2. Hematopoietic Stem Cell Survival

The first evidence for function of VEGF as an intracrine signalling factor came through investigating its role in hematopoietic stem cell survival [10]. Knockout of VEGF in bone marrow mononuclear cells inhibited their ability to restore the hematopoietic compartment in lethally-irradiated mice. VEGF-deficient hematopoietic stem cells fail to form colonies in vitro and exhibit morphological characteristics associated with apoptosis. Soluble VEGFR1 administration to mice failed to induce hematopoietic stem cell apoptosis, whereas the cell permeable VEGFR kinase inhibitors ZD4190 and SU5416 reduce in vitro colony formation, suggesting that an internal autocrine loop is active and the survival activity of VEGF is not accessible to inhibitors that act in the extracellular compartment. Enforced internal expression of VEGF or the alternative VEGFR1 ligand PlGF rescued the in vitro colony formation and in vivo repopulation potential of VEGF-deficient hematopoietic stem cells. This suggests a novel VEGFR1-dependent mechanism in intracrine VEGF-mediated hematopoietic stem cell survival, in addition to the requisite role for VEGFR2 during haematopoietic differentiation in vivo [31].

### 2.3. Endothelial Cell Survival and Metabolism

Intracrine VEGF has also been implicated in endothelial cell homeostasis and survival. Endothelial-specific knockout of VEGF in *VE-CAD-Cre; Vegfa^fl/fl^* mice leads to progressive endothelial degeneration in adults, with fatigue and sudden death often occurring by 25 weeks of age [17]. These mice display systemic vascular pathologies, haemorrhaging, microinfarcts, and chronic inflammation, with an increase in apoptotic endothelial cells causing ruptured endothelial lining and acting as a procoagulant. The level of VEGF in serum, as well as expression in whole tissue or organ samples was indistinguishable between wildtype and *VE-CAD-Cre; Vegfa^fl/fl^* mice, indicating that the systemic vascular dysfunction cannot be rescued by paracrine VEGF, implicating a cell-autonomous role. In vitro culture of VEGF-deficient endothelial cells demonstrated increased apoptosis, particularly in response to cell stress such as hypoxia, and this could not be ameliorated by addition of exogenous VEGF, or co-culture with wildtype cells. Blockage of extracellular VEGF with the neutralising antibody bevacizumab failed to block VEGFR2 phosphorylation, whereas the intracellular inhibitor SU4312 substantially neutralised intracellular phosphorylation, suggesting phosphorylation of VEGFR2 occurs intracellularly in the absence of VEGF secretion, and implying an intracrine role for VEGF in endothelial cell survival. The role of endothelial VEGF in cell survival and stress response was further interrogated by exposing *VE-CAD-Cre; Vegfa^fl/fl^* mice to intermittent hypoxia [19]. This increased the severity of the systemic vascular defects observed by Lee et al. [17], suggesting that cellular stress has an influence on the phenotype. Knockdown of VEGF with siRNA in HUVECs caused an increase in cell death, and this was not rescued by addition of recombinant VEGF. Similarly, HUVECs treated with high doses of extracellular VEGF neutralising antibody remained viable, whereas treatment with intracellular inhibitor SU4312 caused a drastic reduction in cell confluence. Interestingly, classical indicators of apoptosis, such as cleaved-caspase 3, PARP and TUNEL were not detected in VEGF knockdown endothelial cells; however, drastic mitochondrial fragmentation was observed with concomitant reduction in metabolic activity and depressed mitochondrial function, suggesting intracrine VEGF maintains normal metabolism in endothelial cells. An increase in autophagic vacuoles was observed both in VEGF knockdown HUVECs and in endothelial cells of *VE-CAD-Cre; Vegfa^fl/fl^* mice. Blocking the autophagic pathway rescued cell death in VEGF knockdown HUVECs, suggesting the autophagy pathway is a major contributor to the VEGF knockdown phenotype. FOXO1 has been previously linked to autophagy, metabolism and VEGF signalling, and indeed FOXO1 was upregulated in endothelial cells upon VEGF knockdown. Double-knockdown of VEGF and FOXO1 reduced the cell death and autophagic vacuoles induced by VEGF knockdown, suggesting a transcriptional relationship between VEGF and FOXO1; however, mechanistically this remains to be explored.

### 2.4. Osteoblast Differentiation

Intracrine VEGF also plays an important role in regulating osteoblast differentiation. Knockout of VEGF specifically in osteoblasts causes a reduction in trabecular bone and bone density in *Osx-Cre; Vegfa^fl/fl^* mice, with a resultant increased number of adipocytes in bone marrow [20]. This suggests a switch from bone formation to adipocyte differentiation, which is a hallmark feature of osteoporosis. Culture of bone marrow cells from *Osx-Cre; Vegfa^fl/fl^* mice revealed a reduction in the number of osteoblast colony forming units, and an increase in adipocyte numbers. Addition of recombinant VEGF or extracellular neutralising VEGF antibodies had no effect on cell differentiation; however, retrovirus-mediated overexpression of VEGF164 was able to rescue the defects due to loss of VEGF expression, i.e., increase osteoblasts and reduce adipocytes, implying an intracrine function for VEGF. *Osx-Cre* specific knockout of VEGFR1 and VEGFR2 also reduced the numbers of osteoblasts, but had no effect on increasing adipocyte numbers, suggesting that both receptors are required for the effect of VEGF on osteoblast formation, but not for repression of adipocyte differentiation. Subcellular localisation of VEGF and VEGFRs in bone marrow stem cells reveals VEGF and VEGFR2 are detected in the cytoplasm and nucleus, whereas VEGFR1 is mainly nuclear with the predominant isoform expressed being soluble sVEGFR1. This raises the possibility that sVEGFR1 acts to concentrate VEGF in the perinuclear region, further supporting a role for nuclear VEGF in intracrine function. The authors propose a genetic link between the nuclear envelope protein Lamin A and VEGF, as Lamin A knockout mice exhibit a similar decline in bone mass. Loss of Lamin A in bone marrow stem cells causes a reduction in VEGF expression, suggesting Lamin A is upstream of VEGF and acts to promote its expression. How intracrine VEGF regulates the balance between osteoblast and adipocyte differentiation remains to be mechanistically explored, but possibly involves VEGF-mediated stimulation of RUNX2 to promote osteoblastogenesis and repression of PPARγ2 to inhibit adipogenesis.

## 3. Intracrine VEGF in Cancer

VEGF has commonly been implicated in cancer progression through its classical role in inducing angiogenesis and enhancing tumour vascularisation. However, the failure of some anti-angiogenic therapies in reducing cancer burden has raised the possibility that VEGF plays additional non-angiogenic functions in cancer growth and survival [32,33]. There is now a growing body of evidence pointing to an intracrine role for VEGF in a variety of different cancers, which will no doubt have impacts on the choice of anti-VEGF therapies used in the clinic [34].

### 3.1. Acute Myeloid Leukemia

An internal role for VEGF signalling was first proposed in acute myeloid leukemia through the discovery that phosphorylated VEGFR2 was predominantly localised to the nucleus in these cells [21]. Inhibition of VEGFR2, either by a cell-permeable kinase inhibitor, or anti-VEGF antibody treatment that acts externally, induced apoptosis in AML cells; however, the intracellular kinase inhibitor had a much stronger proapoptotic effect. Interestingly, anti-VEGF antibody treatment reduced nuclear localisation of VEGFR2 and shifted its localisation to the cell surface, whereas the kinase inhibitor had little effect on the nuclear localisation of VEGFR2. This suggests that autocrine release of VEGF from AML cells is required to then act on VEGFR2 to promote its nuclear localisation, and thus VEGF may not be functioning as a true intracrine in this instance. Importantly, the different modes of VEGF inhibition affected distinct signalling pathways in the cell, with internal inhibition decreasing ERK1/2 and AKT phosphorylation, whereas external inhibition decreased NF-kB activation and DNA binding. When AML cells were treated with both inhibitors, a synergistic pro-apoptotic effect was observed, and cells were also more susceptible to the chemotherapeutic etoposide. This suggests that both internal and external modes of VEGF inhibition may be beneficial to cancer therapy.

### 3.2. Multiple Myeloma

A similar observation was found in multiple myeloma cells, where VEGFR1 is predominantly localised to the nucleus, rather than the cell surface [22]. Neutralising antibodies directed against VEGFR1 suppressed the growth of these cells, and caused VEGFR1 to be localised to the cytoplasm rather the nucleus. While this study did not provide direct evidence that VEGF functions in an intracrine manner, and while an external inhibitor is able to affect VEGFR1 localisation inside the cell, it is nevertheless intriguing that the intracellular trafficking of VEGFR1 suggests a specific role for nuclear VEGFR1 in promoting cell growth.

### 3.3. Renal Cell Carcinoma

A non-angiogenic role for VEGF in renal cell carcinoma has also been discovered, whereby autocrine VEGF promotes cell growth through interaction with Nrp1 [23]. VEGF shRNA knockdown in the renal carcinoma cell line A-498 reduced tumour growth cell-autonomously in a xenograft mouse model, even when control shRNA and VEGF shRNA cells were mixed in equal quantities before engraftment. This demonstrates that paracrine secretion of VEGF from control cells is unable to rescue the growth of VEGF-deficient cells within the same tumour microenvironment, further indicating a role for intracellular VEGF in cell growth, independent of its proangiogenic function.

### 3.4. Breast Cancer

More definitive evidence of a role for intracrine VEGF in cancer came from a study using breast cancer cell lines (MDA-MB-231 and MCF-7) in which VEGF siRNA knockdown induced cell death [24]. Knockdown of VEGFR2 and Nrp1 had no effect on these cells, whereas knockdown of VEGFR1 similarly induced cell death, implicating a VEGF-VEGFR1 interaction in breast cancer cell survival. Exogenous addition of VEGF did not rescue the cell death induced by VEGF knockdown, implying an intracrine role. Further to this, extracellular VEGFR1 blocking antibodies, VEGF blocking antibodies or soluble sVEGFR1 addition did not affect cell survival. Localisation studies revealed VEGFR1 is predominantly localised to the nucleus where is co-localises with the nuclear envelope protein Lamin A. The function of VEGF-VEGFR1 in the nucleus and its mechanistic role in promoting cell survival remains to be explored, but this study adds to the growing body of evidence suggesting nuclear localisation is key to the intracrine function of VEGF.

### 3.5. Skin Cancer

Other studies have noted the differential susceptibility of cancer cells to intracellular versus extracellular VEGF inhibitors in support of an intracrine role for VEGF in cell growth or survival. Mouse models of skin cancer and human squamous cell carcinoma cell lines showed a stronger inhibition of cell proliferation in response to the intracellular VEGFR inhibitors Sunitinib and BI-1120 compared to external neutralising antibodies and blocking peptides targeting VEGFR1 [25]. This suggests that autocrine intracellular VEGFR signalling may be contributing to cell proliferation in these cancers, and that intracellular inhibitors may provide more effective therapy.

### 3.6. Extrahepatic Bile Duct Cancer

Another study examined the response of extrahepatic bile duct cancer cell lines to exogenous VEGF and internal versus external VEGFR inhibitors [26]. Recombinant VEGF treatment induced proliferation of cells, and this was diminished by treatment with external neutralising antibodies to VEGF, VEGFR1, and VEGFR2, but more significantly supressed by the intracellular VEGFR inhibitors TKI-II and Apatinib. While this exogenous effect of VEGF is not considered intracrine, the authors found that such VEGF treatment induced nuclear accumulation of phosphorylated VEGFR1 and VEGFR2, and also enhanced VEGF promoter activity, increasing VEGF mRNA and protein levels, thus suggesting an internal autocrine VEGF loop. These cells (FRH-0201) were also injected into NOD/SCID mice, where Apatinib treatment decreased tumour growth, while only mildly reducing blood vessel density in the tumour, suggesting the effects of Apatinib are at least partially angiogenesis-independent.

### 3.7. Colorectal Cancer

A number of studies have investigated the role of intracrine VEGF in colorectal cancer growth, survival and invasion. Knockout of VEGF by homologous recombination in human colorectal cancer cell lines HCT116 and LS174T causes a significant inhibition in growth and an increase in spontaneous apoptosis, which could not be rescued by addition of recombinant VEGF to the growth media [27]. Treatment with bevacizumab had no effect on the cells, suggesting VEGF is mediating survival via an intracrine mechanism. VEGF knockout or knockdown with siRNAs also increased the sensitivity of these cell lines to the chemotherapeutic drug 5-flurouracil. VEGFR1 is the predominant receptor expressed in these cells, however VEGFR1 kinase inhibition with SU5416 had no effect on cell death, suggesting that VEGFR1 kinase activity is not required for mediating survival of these cells. A follow-on study identified decreased phosphorylation of AKT, ERK1/2, PRAS40, and p70S6K in colorectal cancer cells upon VEGF and VEGFR1 siRNA knockdown [28]. Adding recombinant VEGF or bevacizumab to the growth media did not alter the phosphorylation of these signalling proteins, and neither did VEGFR1 neutralising antibody treatment or kinase inhibition with pazopanib or SU5416. Therefore, this reinforces a role for intracrine VEGF and VEGFR1 in colorectal cancer cells, independent of VEGFR1 kinase activity. Subcellular localisation studies revealed the strongest co-localisation of VEGF and VEGFR1 was observed on or near the nuclear membrane. To interrogate the mechanism of VEGF knockdown-induced AKT dephosphorylation, cells were treated with phosphatase inhibitors. While AKT is typically phosphorylated on serine/threonine residues, the Ser/Thr phosphatase inhibitor okadaic acid failed to rescue AKT phosphorylation in VEGF knockdown cells. Rather, treatment with the tyrosine phosphatase inhibitor sodium vanadate rescued AKT phosphorylation, suggesting that AKT may be regulated by the inactivation of upstream signalling molecules, such as other receptor tyrosine kinases. In fact, several receptor tyrosine kinases showed decreased phosphorylation upon VEGF knockdown, including EGFR and c-MET, which were rescued by sodium vanadate treatment. The authors propose that VEGF depletion activates a tyrosine phosphatase that reduces the activity of multiple receptor tyrosine kinases leading to reduced activity of AKT and ERK1/2, and subsequent reduced cell survival and enhanced chemosensitivity. Further work also investigated the effect that loss of VEGF has on colorectal cancer cell migration and invasion in in vitro transwell assays [29]. VEGF siRNA knockdown in HCT116, SW480, SW620 and HT29 cells significantly reduced cell migration towards a foetal bovine serum attractant. This assay was also performed with additional tumour cell types such as gastric, ovarian and breast, which showed a similar reduction in migration, suggesting the role of VEGF in cell migration is not unique to colorectal cancer. Colorectal cancer cells HCT116 and SW480 also had lower rates of invasion into a Matrigel coated transwell upon VEGF siRNA knockdown. In both migration and invasion assays, treatment of cells with bevacizumab had no detrimental effect. In fitting with previous data, VEGFR1 siRNA knockdown strongly inhibited cell migration, whereas no effect was observed with VEGFR1 kinase inhibition, further supporting a kinase independent role. No change in proteins associated with EMT, such as E-cadherin, N-cadherin, Snail, or Zeb1, could account for the changes in cell migration observed. However, a strong reduction in focal adhesion kinase activation, and its upstream regulators (phospho-EGFR and phospho-c-MET) was observed upon VEGF and VEGFR1 siRNA knockdown. Overexpression of c-MET in VEGF siRNA knockdown cells partially rescued the migratory defects, however overexpression of focal adhesion kinase or inhibiting tyrosine phosphatases with sodium vanadate was detrimental even to control siRNA treated cells. Taken together, intracrine VEGF clearly plays an important role in colorectal cancer cell survival and cell migration, and this is likely mediated through a non-kinase function of intracellular VEGFR1. How the VEGF-VEGFR1 intracrine complex mediates downstream signalling remains to be deciphered, but may involve modulation of other receptor tyrosine kinases and their cognate signalling cascades, or modulation of unknown tyrosine phosphatases that in turn regulate other receptors or signalling molecules.

### 3.8. Adenocarcinoma

An interaction between MET and intracrine VEGF signalling has also been implicated in adenocarcinoma [30]. The epithelial cancer cell lines H441 and PC3 strongly co-express MET and VEGFR2, however stimulation of MET with HGF supresses VEGFR2 protein levels, while increasing VEGF levels. Inactivation of MET via PI3K inhibition blocks both the increase in VEGF levels and the decrease in VEGFR2 levels induced by HGF. The authors questioned whether there may be a direct link between VEGF induction and VEGFR2 suppression. Overexpression of a VEGF cDNA within the cell reversed the increase in VEGFR2 seen upon MET disruption, whereas addition of exogenous VEGF had no effect. Likewise, knockdown of VEGF with siRNAs increased baseline levels of VEGFR2, whereas treatment of cells with anti-VEGF antibody did not. Furthermore, disruption of clathrin-mediated endocytosis did not affect VEGFR2 modulation by MET. Collectively, this evidence points to an intracrine role for VEGF in supressing VEGFR2, independent from extracellular VEGF. Proximity ligation assays confirmed an intracellular interaction between VEGF and VEGFR2 that co-localises to the endoplasmic reticulum. Intracellular VEGF phosphorylates VEGFR2, and interrogation with the proteasomal inhibitor MG132 and siRNA knockdown of several E3-ligases discovered that HGF treatment induces Cbl-independent K48 ubiquitination of VEGFR2 by HRD1 and gp78 which triggers ER-associated degradation supported by the unfolded protein response factor IRE1α. Whether the MET-induced phosphorylation of VEGFR2 by intracrine VEGF is required for ubiquitination remains to be investigated, but provides an intriguing hypothesis for how intracrine VEGF may function mechanistically.

## 4. Potential Mechanisms of Intracrine VEGF Function

**Figure 2:**
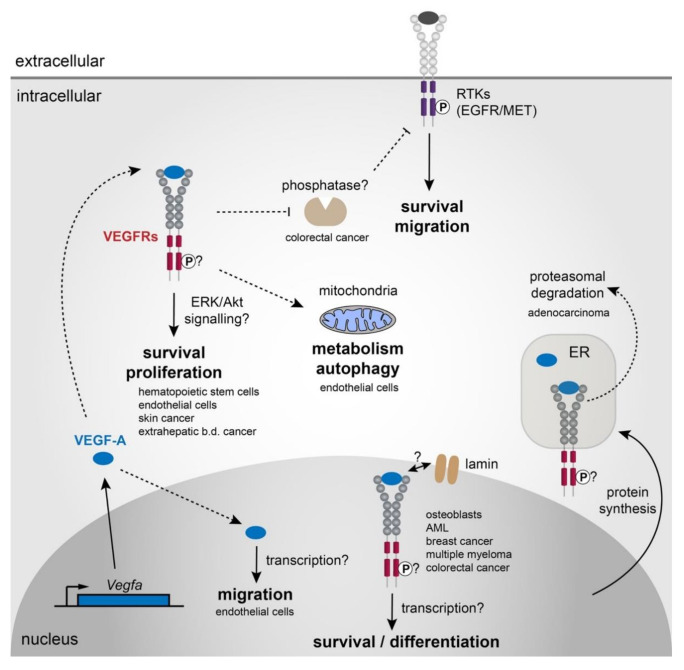
Potential mechanisms of intracrine VEGF-A signalling. VEGF-A may act via several context and cell-type dependent mechanisms to control diverse aspects of cell behaviour such as survival, migration, metabolism and differentiation. Evidence suggests VEGF-A may bind to one or more of its receptors (VEGFR1, VEGFR2, and Nrps) within the cell to regulate survival and proliferation. Whether these receptors are localised to the cytoplasm or within particular sub-cellular compartments, organelles or vesicles remains to be determined and is likely context specific. Phosphorylation and activation of these receptors intracellularly may elicit responses via downstream mediators such as ERK or Akt, but in some instances, intracrine signalling does not appear to be dependent on the tyrosine kinase activity of the VEGFRs. Intracrine VEGF-A has also been shown to regulate mitochondrial metabolism and autophagy; however; the cellular mechanisms remain to be uncovered. Another study suggested intracrine VEGF-A may regulate an unknown tyrosine phosphatase, that in turn regulates signalling via other RTKs such as EGFR or MET. VEGF-A and/or VEGF-A/VEGFR complexes also translocate to the nucleus to mediate aspects of intracrine signalling, suggesting they may play a role in regulating transcription. Studies have also suggested an interaction with the nuclear envelope protein Lamin may be important. Interaction of VEGF-A with VEGFR2 in the endoplasmic reticulum has been shown to promote degradation of VEGFR2 via ubiquitin-mediated proteasomal degradation, which may provide a mechanism for intracrine VEGF-A signalling triggers ER-associated degradation supported by the unfolded protein response factor IRE1α. Whether the MET-induced phosphorylation of VEGFR2 by intracrine VEGF is required for ubiquitination remains to be investigated, but provides an intriguing hypothesis for how intracrine VEGF may function mechanistically.

In contrast to its functions outside of the cell, how VEGF mechanistically functions in an intracrine manner to modulate cell survival, growth, and differentiation remains to be fully characterised, and is likely to be context and cell-type dependent. Potential intracrine signalling mechanisms based on the aforementioned studies are summarised in Figure 2.

### 4.1. A Role for VEGF/VEGFR in the Nucleus?

Several lines of evidence point toward an important role for VEGF and VEGFR1/2 in the nucleus [18,20,21,22,24,26,28]. A key outstanding question is how VEGF is able to translocate to the nucleus. VEGF contains a stretch of basic amino acids corresponding to exon 6a of the transcript that may act as a potential nuclear localisation sequence (NLS); however, due to alternative splicing, this potential NLS is present in VEGF189, but not in the shorter VEGF121 or VEGF165 isoforms. Consistent with this, transfection of MCF-7 cells with a tagged VEGF189 construct demonstrates nuclear localisation, whereas tagged VEGF121 and VEGF165 constructs remain localised in the cytoplasm [35]. However, if the NLS in VEGF is important for intracrine function, then how is intracellular overexpression of VEGF165 able to rescue many of the effects of VEGF knockout/knockdown in the various examples described above? These findings suggest that either a nuclear role for VEGF is not essential for intracrine function, or that perhaps additional co-factors associate with and translocate VEGF into the nucleus rather than reliance on a cis-acting NLS. Characterisation of the importance of nuclear VEGF will require further detailed exploration of the specific VEGF isoforms required for intracrine signalling, functional interrogation and mutagenesis of the NLS sequence(s), and modulating nuclear import/export to assess outcomes for VEGF intracrine signalling. It may also be more beneficial to internally overexpress the VEGF189 isoform, rather than the more abundant VEGF165, to elicit intracrine effects. Other alternative VEGF isoforms have also shown nuclear localisation, such as the anti-angiogenic splice variant VEGF165b in the human foetal eye [36], and the long 180 amino acid N-terminal extension of L-VEGF, which is cleaved and translocates to the nucleus upon stimulation with hypoxia [37]. Whether these alternative VEGF isoforms play any role in intracrine signalling remains to be investigated.

Interestingly, VEGF-D is known to accumulate in the nuclei of lung fibroblasts where it associates with RNA Polymerase II and c-Myc, suggesting that VEGF-D may be involved in regulating transcription [38]. Given the similarity between VEGF-A and other VEGF family members, it is feasible that VEGF-A may also interact with transcription machinery in a similar manner and should be investigated as a potential molecular mechanism of VEGF intracrine function. Indeed, characterising the binding partners of VEGF-A within the cell may also provide new insight to how intracrine VEGF signalling may be regulated.

As well as VEGF, the VEGF receptors VEGFR1 and VEGFR2 demonstrate nuclear localisation under some circumstances [20,21,22,24,26,28]. It will be important to determine whether this nuclear localisation of VEGFRs is important for the intracrine signalling response, and how VEGFRs functionally act in the nucleus. VEGFR2 has been shown to rapidly shuttle between the nucleus and cytoplasm in HUVECs; however, a point mutation at tyrosine 951 inhibits this nuclear-cytoplasmic shuttling, suggesting phosphorylation at this tyrosine residue may be essential [39]. Further, co-immunoprecipitation of VEGFR2 from HUVEC nuclear extracts revealed an interaction between VEGFR2 and the transcription factor Sp1, which together bind to a Sp1-responsive element in the *Vegfr2* promoter, suggesting VEGFR2 can regulate its own transcription in a positive feedback loop. This raises several important questions. Is phosphorylation of VEGFRs required to elicit intracrine function, for example, targeting to the nucleus? Is VEGFR kinase activity also required? In some examples described above, VEGFR kinase inhibitors were used to demonstrate the necessity of VEGFR kinase activity for intracrine signalling function [10,17,19,21,25,26], whereas in other examples kinase activity was dispensable [27,28]. It will be important to determine the necessity of VEGFR kinase activity in the intracrine signalling response, which will likely be context and cell-type specific. Furthermore, given the interaction of VEGFR2 with Sp1, it will be important to determine if this complex interacts with additional promoter elements to control gene transcription, and if this is a molecular mechanism underlying VEGF intracrine signalling function. In addition, it will be important to determine what role additional VEGF receptors may play in the intracrine signalling pathway, such as NRP1 which likely mediates the intracrine VEGF function in renal cell carcinoma [23]. It is also feasible that intracrine VEGF signalling occurs independently of classical VEGF receptors altogether, and that intracellular VEGF interacts with other as yet unknown proteins or complexes within the cell to elicit intracrine signalling responses.

### 4.2. A Role for VEGF/VEGFR in Other Sub-Cellular Compartments?

VEGF and/or VEGFRs may also activate intracrine signalling via additional sub-cellular compartments other than the nucleus. VEGFRs that are stimulated with external ligands at the cell membrane are subsequently internalised in endosomes which is necessary for downstream signalling activity [40]. In HUVECs, for example, VEGFR2 internalisation upon ligand binding does not terminate the signalling activity of the receptor, but rather this can continue in endosomes [41]. Is it possible that intracellular VEGF could also access and signal via VEGFR2 located in endosomes, thus mediating an intracrine signalling event? Newly translated intracellular VEGF has also been shown to interact with VEGFR2 in the endoplasmic reticulum, mediating the intracrine function of VEGF in adenocarcinoma cells [30]. It will be important to determine if VEGF also plays similar roles in the endoplasmic reticulum, or additional organelles, in other cellular contexts of intracrine signalling.

In most cases, intracrine VEGF has been implicated in regulating cell growth and survival. How intracrine VEGF molecularly activates pro-survival pathways remains to be explored. As well as inhibiting apoptosis, intracrine VEGF has also been implicated in preventing autophagy and altering mitochondrial metabolism [19]. Whether this is via a direct interaction or localisation of VEGF to the mitochondria, or as a consequence of a transcriptional role for VEGF in the nucleus, for example, will be worthy of investigation.

## 5. Conclusions

Intracrine VEGF signalling is now emerging as an important intracellular pathway regulating cell growth and survival. While an intracrine function for VEGF has been documented in an ever-increasing variety of cell-types and cancers, the molecular mechanisms underpinning intracrine VEGF function have remained poorly defined. Evidence is emerging for the roles of VEGFRs in mediating VEGF intracrine signalling, in addition to their classical roles as cell surface receptors for extracellular ligands. Whether VEGFR kinase activity is required for VEGF intracrine signalling appears to be cell-type specific, implying VEGFRs or VEGF itself may have additional intracellular binding partners mediating intracrine signalling events and functional outcomes. Understanding the molecular mechanisms involved in VEGF intracrine signalling will be of great interest, especially in regards to developing targeted therapies for cancers and other diseases in which intracrine VEGF signalling has been shown to play a role.

## Figures and Tables

**Table 1:** Examples of VEGF-A acting in an intracrine manner.

Cell Type	Cell Response	Potential Mechanism	Reference
Endothelial/ACE cells (in vitro)	Migration/wound healing	Binding VEGFR2/nuclear localisation	Li et al., 2000 [18]
Bone marrow mononuclear cells (in vivo and in vitro)	Haematopoiesis	Binding VEGFR1/VEGFR2 and TK signalling	Gerber et al., 2002 [10]
Endothelial (in vivo and in vitro)	Survival	Binding VEGFR2, mitochondrial dysfunction and autophagy	Lee et al., 2007 [17] Domigan et al., 2015 [19]
Osteoblast (in vivo and in vitro)	Cell fate and differentiation	Binding VEGFR1/VEGFR2 and nuclear localisation	Liu et al., 2012 [20]
AML/primary cells, Hel and HL-60 (in vitro)	Survival	Binding VEGFR2 and nuclear localisation	Santos et al., 2004 [21]
Multiple myeloma/ primary patient derived cells (in vitro)	Proliferation and migration	Binding VEGFR1 and nuclear localisation	Vincent et al., 2005 [22]
Renal carcinoma/A-498 (in vivo and in vitro)	Proliferation	Binding to NRP1	Cao et al., 2012 [23]
Breast cancer/ MDA-MB-231 and MCF-7 (in vitro)	Survival	Binding VEGFR1 and nuclear localisation, but not TK activity dependent	Lee et al., 2007 [24]
Skin cancer and squamous cell carcinoma/K5 SOS mouse model (in vivo)	Proliferation	Binding to VEGFR and TK signalling	Lichtenberger et al., 2010 [25]
Extrahepatic bile duct/FRH-0201 and EGI-1 (in vivo and in vitro)	Proliferation	Binding to VEGFR and TK signalling	Peng et al., 2016 [26]
Colorectal cancer/HCT116 and LS174T (in vitro)	Survival	Binding to VEGFR1 but not TK activity dependent. Activation of a tyrosine phosphatase.	Samuel et al., 2011 [27] Bhattacharya et al., 2016 [28]
Colorectal cancer/HCT116 and SW480 (in vitro)	Survival and migration	Binding to VEGFR1 but not TK activity dependent.	Bhattacharya et al., 2017 [29]
Adenocarcinoma/H441 and PC3 (in vitro)	Proliferation	Binding to VEGFR2 in the ER to regulate its stability.	Chen et al., 2015 [30]
